# Puerperal Fever and Erysipelas

**Published:** 1857-07

**Authors:** J. Levergood

**Affiliations:** Wrightsville, Penn.


ART. III.—*Puerperal Fever and Erysipelas.* By J. LEVERGOOD, M.D., Wrightsville, Pa.

Puerperal fever is a disease peculiarly characterized by its insidious mode of attack, the rapidity with which it proves fatal, and the difficulty experienced in compelling it to succumb to treatment. In these respects the whole category of diseases to which the parturient female is liable, may be searched in vain to find its counterpart. In regard to its nature, the most discordant and antagonistic views have been held, and, consequently, the treatment has been as diversified as the opinions entertained of its pathological character. This malady made its appearance not long since in the practice of a physician of this place, and as its commencement, progress, and termination, clearly indicated its origin, I will, briefly, with the kind concurrence of the physician to whom allusion is made, relate the circumstance as it occurred.

In the month of March, 1853, Dr. B. C. Lloyd, while professionally attending, in this place, for phlegmonous erysipelas, involving the entire left arm, a man whose system was very much debilitated by the excessive use of ardent spirits, was summoned to Mrs. D., in labor with her third child. This lady lived more than a mile from town, in what is known as the "York Valley," renowned for its beautiful farms, fertile soil, and wealthy inhabitants, and proverbial for its healthfulness. At the time his attendance upon her began, there was no prevailing epidemic disease of any kind whatever, and Mrs. D. was enjoying perfect health. An examination showed the vertex to be the presenting part, and her labor terminated as easily and speedily as usual in such cases. On the third day, puerperal fever symptoms, one of the most prominent of which was abdominal tympanitis, developed themselves, and two days afterwards she expired.

The second case was that of Mrs. S. This was her sixth accouchement, and, like all the previous ones, it ended as favorably as an entirely natural labor in a female who, at the time of and previous to her confinement, was in the enjoyment of the most robust health, could be expected to end. She, also, lived in the "Valley," some two miles from town, and about one mile from the residence of Mrs. D. Symptoms of puerperal fever manifested themselves on the third day subsequent to delivery, and, notwithstanding the prompt assistance of a physician from York, all efforts to save her life proved unavailing.

The third case was that of Mrs. N., also the wife of a Valley farmer, and living fully five miles from town. This was not her first parturition, but the number of children she has had the writer is not aware of. The whole course of the labor differed in no material respect, except in its being a twin case, from those I have mentioned. She, also, was attacked by the disease, and, although, as in the previous cases, active and decided antiphlogistic treatment was promptly employed, the case had a fatal termination.

Previous to his attendance upon the case of phlegmonous erysipelas, there had been in the charge of Dr. Lloyd, both in town and country, a number of midwifery cases, and, in no single instance, did peritonitis make its appearance; but from the time he took charge of the case mentioned, until he relinquished it, embracing a period of three weeks, he lost every parturient female, viz., the three I have mentioned, to whom he was called. The writer was engaged in obstetrical practice at the same time, some cases of which were not very remote from those of Dr. Lloyd, but in none did the disease make its appearance. It therefore appearing very evident that the case of phlegmonous erysipelas was the source from which emanated the *materies morbi* that was proving so disastrous to the childbed women under his care, and that he was the medium through which it was disseminated, the doctor at once relinquished any further connection with the case. The writer being then solicited to take charge of the man, positively declined to do so, feeling morally certain that his patients would share the same fate as those of his friend; the service of a physician from a neighboring village upon the opposite side of the river were secured, and from that time, no other cases of the disease appeared.

*Post hoc, ergo propter hoc*, is, we are all aware, a very fallacious and illogical method of reasoning, but the rise, progress, and termination of this disease, were attended by circumstances too palpable to admit of explanation upon the supposition of their being merely a succession of coincidences. The intimate pathological connection existing between puerperal fever and erysipelas, and the possession by these diseases of the power of mutually reproducing each other, are questions which frequent observation has so abundantly confirmed, as to place them without the bounds of reasonable conjecture. One of the first of living accoucheurs, says, "that when the fingers of medical men were impregnated with the morbid secretions thrown out in erysipelatous inflammation, the inoculation of these matters into the genital canals of parturient females produced puerperal fever in them, in the same way as the inoculation of the secretions from patients who had died of puerperal fever itself;"* and were it necessary to amplify quotations to the same effect, it might be done to an almost unlimited extent.

* Simpson's Obstetric Memoirs, vol. ii, p. 43.

